# Implementation of a 7T Epilepsy Task Force consensus imaging protocol for routine presurgical epilepsy work-up: effect on diagnostic yield and lesion delineation

**DOI:** 10.1007/s00415-023-11988-5

**Published:** 2023-10-07

**Authors:** Gilbert Hangel, Gregor Kasprian, Stefanie Chambers, Lukas Haider, Philipp Lazen, Johannes Koren, Robert Diehm, Katharina Moser, Matthias Tomschik, Jonathan Wais, Fabian Winter, Vitalij Zeiser, Stephan Gruber, Susanne Aull-Watschinger, Tatjana Traub-Weidinger, Christoph Baumgartner, Martha Feucht, Christian Dorfer, Wolfgang Bogner, Siegfried Trattnig, Ekaterina Pataraia, Karl Roessler

**Affiliations:** 1 Department of Neurosurgery, Medical University of Vienna, Währinger Gürtel 18-20, 1090 Vienna, Austria; 2 Department of Biomedical Imaging and Image-Guided Therapy, High Field MR Centre, Medical University of Vienna, Vienna, Austria; 3 Christian Doppler Laboratory for MR Imaging Biomarkers, Vienna, Austria; 4 Medical Imaging Cluster, Medical University of Vienna, Vienna, Austria; 5 Division of Neuroradiology and Musculoskeletal Radiology, Department of Biomedical Imaging and Image-Guided Therapy, Medical University of Vienna, Vienna, Austria; 6 NMR Research Unit, Faculty of Brain Science, Queens Square MS Centre, Department of Neuroinflammation, UCL Queen Square Institute of Neurology, University College London, London, United Kingdom; 7 Department of Radiology and Nuclear Medicine, VU University Medical Centre, Amsterdam, The Netherlands; 8 Department of Neurology, Klinik Hietzing, Vienna, Austria; 9 Center for Rare and Complex Childhood Onset Epilepsies, Member of ERN EpiCARE, Department of Pediatrics and Adolescent Medicine, Medical University of Vienna, Vienna, Austria; 10 Department of Neurology, Medical University of Vienna, Vienna, Austria; 11 Division of Nuclear Medicine, Department of Biomedical Imaging and Image-Guided Therapy, Medical University of Vienna, Vienna, Austria

**Keywords:** 7 T MRI, Epilepsy, 7 T Epilepsy Task Force consensus recommendation, Ultra-high-field

## Abstract

**Objective:**

Recently, the 7 Tesla (7 T) Epilepsy Task Force published recommendations for 7 T magnetic resonance imaging (MRI) in patients with pharmaco-resistant focal epilepsy in pre-surgical evaluation. The objective of this study was to implement and evaluate this consensus protocol with respect to both its practicability and its diagnostic value/potential lesion delineation surplus effect over 3 T MRI in the pre-surgical work-up of patients with pharmaco-resistant focal onset epilepsy.

**Methods:**

The 7 T MRI protocol consisted of T1-weighted, T2-weighted, high-resolution-coronal T2-weighted, fluid-suppressed, fluid-and-white-matter-suppressed, and susceptibility-weighted imaging, with an overall duration of 50 min. Two neuroradiologists independently evaluated the ability of lesion identification, the detection confidence for these identified lesions, and the lesion border delineation at 7 T compared to 3 T MRI.

**Results:**

Of 41 recruited patients > 12 years of age, 38 were successfully measured and analyzed. Mean detection confidence scores were non-significantly higher at 7 T (1.95 ± 0.84 out of 3 versus 1.64 ± 1.19 out of 3 at 3 T, p = 0.050). In 50% of epilepsy patients measured at 7 T, additional findings compared to 3 T MRI were observed. Furthermore, we found improved border delineation at 7 T in 88% of patients with 3 T-visible lesions. In 19% of 3 T MR-negative cases a new potential epileptogenic lesion was detected at 7 T.

**Conclusions:**

The diagnostic yield was beneficial, but with 19% new 7 T over 3 T findings, not major. Our evaluation revealed epilepsy outcomes worse than ILAE Class 1 in two out of the four operated cases with new 7 T findings.

**Supplementary Information:**

The online version contains supplementary material available at 10.1007/s00415-023-11988-5.

## Introduction

### Focal epilepsy

Epilepsy affects over 50 million people worldwide, making the treatment of epilepsy a global challenge for neurology [1]. Focal epilepsies as defined by the International League Against Epilepsy (ILAE) [2], not only causes seizures, but also cognitive, psychological, and social impairments [3]. Up to 40% of these patients do not respond to treatment with two or more anti-seizure medications (ASMs) and are thus termed “pharmaco-resistant,” or “refractory” [4]. In these cases, surgery is recommended [5] and can be curative, provided a complete resection/disconnection of the epileptogenic zone (the area of cortex indispensable for the generation of clinical seizures [6]) can be achieved. Pre-surgical evaluation uses a variety of diagnostic tools, such as an analysis of seizure semiology, (non-) invasive electrophysiological (EEG) recordings, neuropsychological testing, and structural as well as functional neuroimaging. These try to define the location and boundaries of the epileptogenic zone as well as eloquent brain areas. Furthermore, in MRI negative cases even invasive procedures such as implantation of intracranial electrodes for stereo EEG prove to be indispensable [7].

The main pathological entities responsible for focal pharmaco-resistant epilepsies are focal cortical dysplasia (FCD), epilepsy-associated tumors (LEATs, e.g., dysembryoplastic neuroepithelial tumor (DNET) or ganglioglioma (GG)), hippocampal sclerosis (HCS), or vascular malformations [8–10]. Complete resection/disconnection of these lesions may result in seizure freedom in up to 60% of cases [5]. Successful surgery is, however, dependent on the precise localization of epileptogenic foci [6]. The current imaging standard, 3 T MRI [9], does not yield findings in one of three cases (MR-negative, MRN) [10]. In addition to MRN cases, some suspected lesions, including mild cortical dysplasia or epilepsy-associated tumors, are not precisely delineated or optimally characterized on 3 T MRI, and need further invasive EEG exploration. MRN cases have reduced odds for postsurgical freedom from seizures [11], but surgery is still often attempted to reduce seizure frequency, and to prevent the consequences of long-term refractory epilepsies, i.e., cognitive deficits and sudden unexpected death in epilepsy [9].

### 7 T MRI for epilepsy

The higher signal-to-noise ratio and altered contrast behavior of 7 T MRI [12] can increase detection sensitivity and delineation of potential epileptogenic lesions. Consequently, 7 T studies were found to improve FCD detection [13] and better identification of subtle vascular malformations due to increased SWI contrast, as well as delineation of cavernomas [14] and hippocampal scleroses, especially subfield neuronal rarefication correlating with epilepsy outcome [15]. Recent reviews determined that 7 T identified new lesions in 31% of MRN cases [16] and have shown an average detection rate for epileptic foci of 65% compared to 22% at lower fields [17].

Recently, the 7 T Epilepsy Task Force published a consensus protocol recommendation [10] that encompasses the use of sub-millimeter 3D T1-weighted MRI (T1w-MRI), T2-weighted MRI (T2w-MRI), fluid attenuated inversion recovery (FLAIR), and T2*-weighted MRI (T2*w-MRI) sequences of the whole brain. Additional recommended scans are high-resolution coronal T2w-MRI of the hippocampus and white matter (WM)-suppressed imaging (WMS) for the detection of gray matter aberrations.

### Purpose

The purpose of this study was to evaluate the practicality of the 7 T Epilepsy Task Force consensus recommendation in clinical practice and to investigate the advantage of this 7 T protocol compared to a dedicated 3 T MRI routine protocol at a busy epilepsy surgical center. Additionally, we correlated our 7 T images with the sum of clinical diagnostic data, and contextualized to the current state of research on morphological 7 T MRI in epilepsy [10, 16, 17].

## Methods

### Hypothesis

Our hypothesis was that the 7 T Epilepsy Task Force recommendation offers improved rates and confidence for lesion detection, as well as better focal delineation than a dedicated 3 T MRI epilepsy protocol. The null hypothesis accordingly stated that the protocol does not offer any quantifiable benefits over the current clinical gold standard.

### Cohort recruitment

**Fig. 1:**
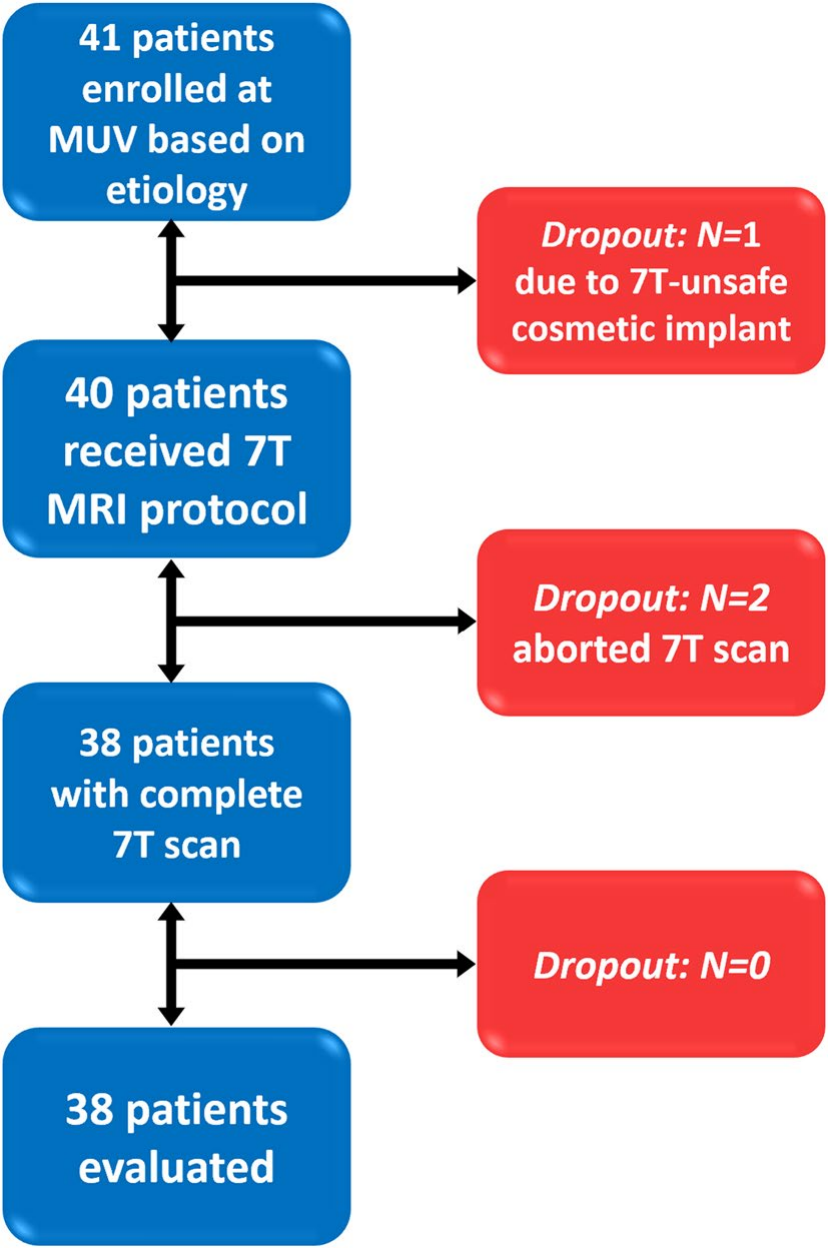
Visualization of subject recruitment and dropouts during measurement and analysis. *7 T*  7 Tesla, *MUV*  Medical University of Vienna

After institutional review board approval (EK 1039/2020) for this prospective study, consecutive patients with pharmaco-resistant focal epilepsy were recruited in 2020–2022 during the pre-surgical evaluation process (according to ILAE standards [6, 18, 19]) at the Medical University of Vienna (MUV) according to the presented inclusion and exclusion criteria by the Department of Neurology, Department of Neurosurgery, and Department of Pediatrics and Adolescent Medicine (Fig. 1).

Inclusion criteria: Age ≥ 12 years; informed consent by the subject, and, if applicable, their legal guardian; pharmaco-resistant focal epilepsy with either suboptimal delineation of the epileptogenic lesion on 3 T MRI or MRN despite the congruency of other pre-surgical investigations (seizure semiology, ictal/interictal video-EEG, neuropsychological testing, PET imaging); and pre-surgical evaluation and/or epilepsy surgery at our centers.

Exclusion criteria: Age < 12 years; weight < 30 kg; claustrophobia; pregnancy; breastfeeding; and ferromagnetic implants for the 7 T scan.

### 7 T Protocol

We conducted all scans on the MUV High-Field MR Centre’s 7 T MR scanner (7 T Magnetom retrofitted to Magnetom.plus, VE12U software, Siemens Healthineers, Erlangen) using a 32-channel head receive / 1-channel transmit coil array (Nova Medical). As a first-generation 7 T scanner, the system is not certified for clinical routine use. Therefore, we were not able to make resection decisions based on 7 T data but compared the data postoperatively.

**Fig. 2:**
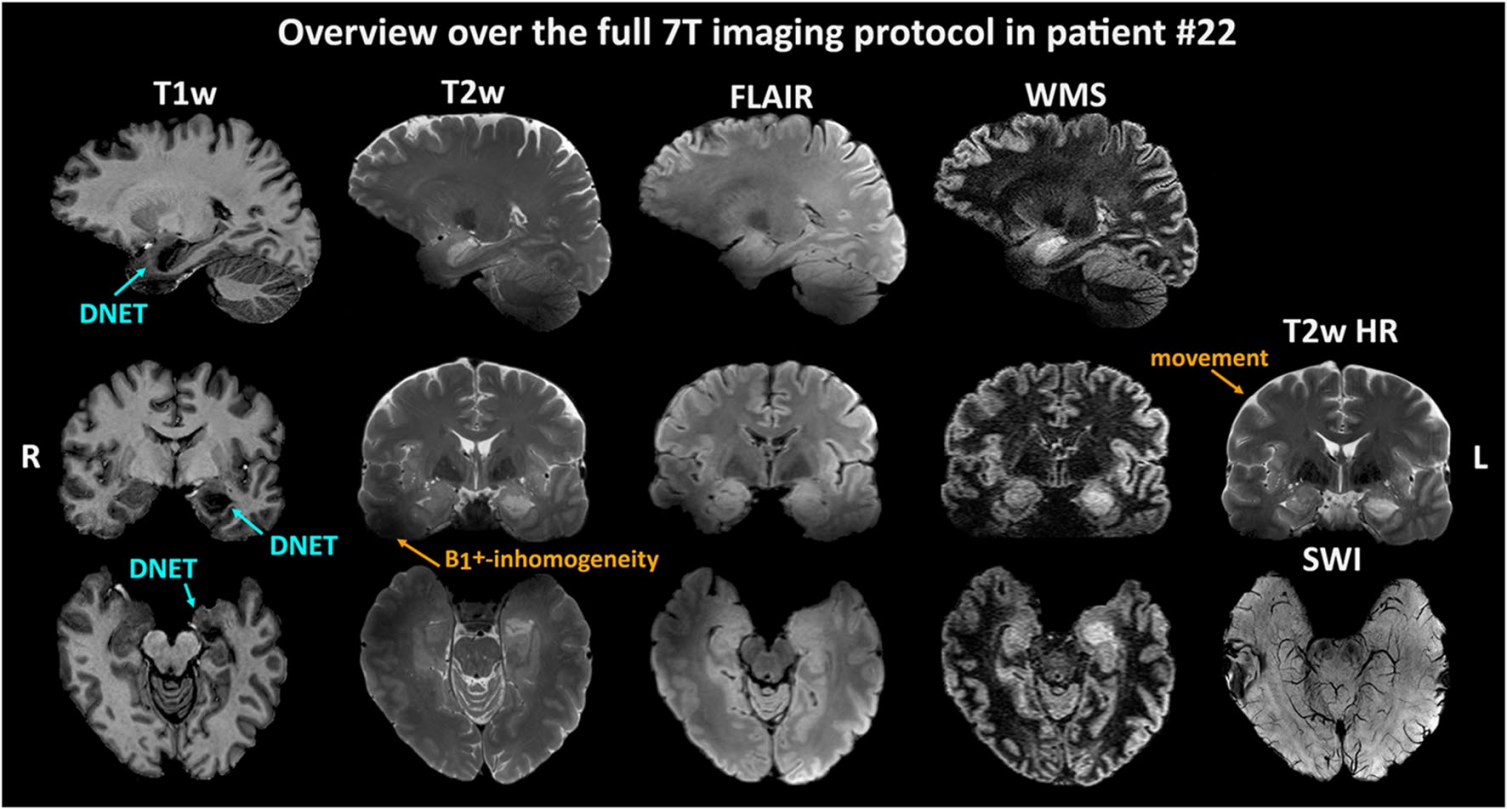
Example of the 7 T epilepsy consensus protocol performance acquired for all modalities in patient #22 with a histologically confirmed dysembryoplastic neuroepithelial tumor (DNET). While 7 T contrast and resolution benefits are quite noticeable, so are radio frequency excitation (B1^+^-)inhomogeneities in particular. Arrows highlight regions of interest for pathology or artifacts. *T1w*  T1-weighted, *T2w* T2-weighted, *FLAIR*  Fluid-attenuated inversion recovery, *WMS*  white matter suppressed, *7 T*  7 Tesla, *SWI*  susceptibility-weighted imaging, *HR*  high resolution, *R*  right side, *L*  left side

The morphological MRI protocol consisted of the following sequences according to the ILAE consensus protocol (more detailed in Suppl. Table 1): 3D T1w (MP2RAGE, TR = 5000 ms, TE = 4.13 ms, T_acq_ = 8:02 min, resolution = 0.75 × 0.75 × 0.75mm^3^); 3D T2w (TSE, TR = 4000 ms, TE = 118 ms, T_acq_ = 7:02 min, resolution = 0.7 × 0.7 × 0.7mm^3^); coronal hippocampal T2w (TSE, TR = 5900 ms, TE = 49 ms, T_acq_ = 6:07 min, resolution = 0.5 × 0.4 × 2.0mm^3^); 3D T2w fluid-suppressed (FLAIR, TR = 8000 ms, TE = 305 ms, T_acq_ = 12:18 min, resolution = 0.9 × 0.9 × 0.9mm^3^); 3D T2w fluid- and WM-suppressed (TSE, TR = 8000 ms, TE = 321 ms, T_acq_ = 3:54 min, resolution = 0.9 × 0.8 × 1.0mm^3^); and transversal SWI (GRE, TR = 21 ms, TE = 14 ms, T_acq_ = 7:29 min, resolution = 0.25 × 0.25 × 1.5mm^3^). Including scan setup times and pre-scans, this imaging protocol required at least 50 min of measurement time. The average image quality is demonstrated in Fig. 2.

### 3 T clinical protocol and other diagnostics

Routine 3 T MRI data were collected as the reference standard from the hospital imaging system for every patient. All 3 Tesla examinations were performed following the Harmonized Neuroimaging of Epilepsy Structural Sequences (HARNESS-MRI) protocol recommended by the ILAE neuroimaging task force [9, 20], with isotropic, millimetric 3D T1w-MRI, FLAIR, and high-resolution 2D sub-millimetric T2w-MRI (detailed in Suppl. Table 1).

3 T MRI results together with Video-EEG monitoring (VEM; semiology at clinical seizure onset, ictal and interictal EEG) and [18F]FDG and MET PET [5, 6] estimated the extent of the epileptogenic zone as the gold standard for further evaluation. Furthermore, if available, resected areas with histological diagnoses as ground-truth, as well as the ILAE outcome classification [21], were collected. All of these data are presented in Table 1.

**Table 1:** Detailed overview over the patient cohort prior to 7 T MRI, including clinical onset monitoring, 3 T MRI, PET, and EEG

Age at 7 T scan	Sex	3 T MRI	Video-EEG	PET	Seizure semiology	Overall preoperative localisation	Resected area	Histology	ILAE Outcome Classification	Outcome time after surgery [months]
32	M	Hippocampal atrophy right	Right and left temporal	Right temporopolar	Not discernible	Right temporal	AMTLR with hippocampectomy right	Hippocampal sclerosis Type 1	Class 1	36
21	M	FCD right frontal	Right frontal	Right frontal	Right hemisphere	Right frontal	Lesionectomy right frontal lobe	FCD 1b	Class 4	36
30	M	MR negative	Not definitively discernible	Right temporolateral dorsal	Right frontal	Right temporolateral	–	–	–	–
26	F	MR negative	Right frontocentral	Right frontolateral	Right frontal	Right frontal	–	–	–	–
31	M	FCD right frontal	Right frontal, left central	Right frontal	Right frontal	Right frontal	Lesionectomy right frontal lobe	FCD 2b	Class 1	27
30	F	Hippocampal asymmetry right	Left frontal	Hippocampal atrophy right	Left frontal	Right temporal	–	–	–	–
14	F	MR negative	Right temporal	Right temporal	Right temporal	Right temporal		–	–	–
43	F	Left temporal	Left temporal	Gyrus temporalis superior left	Left temporal	Left temporal	–	–	–	–
28	F	MR negative	Right parietal/central	Right frontoparietal	Right parietal/central	Right parietal/central	Postcentral parietal resection right, coagulation gyrus postcentralis	Gliosis	Class 4	24
21	M	MR negative	Not definitively discernible	Right insular and right temporolateral	Right frontal	Unclear	–	–	–	–
32	F	MR negative	Bilateral	Negative	Right frontal	Unclear	–	–	–	–
25	F	MR negative	Right hemisphere	Negative	Right hemisphere	Right hemisphere	–	–	–	–
33	F	MR negative	Right frontal and left hemisphere	Negative	Right frontal	Right frontal	–	–	–	–
30	F	FCD left frontal	Left frontal/hemispheric	Left frontal	Not discernible	Left frontal	Left frontal resection	MOGHE	Class 1	32
19	M	Bilat. hippocampal sclerosis	Bilateral temporal	Right temporal	Bilateral Temporal	Bilateral Temporal		–	–	–
20	F	Right temporopolar	Right temporal	Right temporal ventral	Left temporal	Right temporal	Anterior-temporal resection with amygdala hippocampectomy	Mild malformation of cortical development	Class 1	25
24	M	MR negative	Right temporal	Right temporal	Right temporal	Right temporal	AMTLR right	N/A	Class 2	19
31	F	Left parieto-occipital	Left parieto-occipital	left Occipital to temporoparietal	left occipital	Left parieto-occipital	Lesionectomy left angular and parieto-occipital	Polymicrogyria	Class 1	24
49	F	MR negative	Right temporal and left parietal	Bilateral temporal	Right temporal and left parietal	Unclear		–	–	–
35	M	MR negative + falx meningeoma	Left hemisphere	Negative	Left hemisphere	Left hemisphere		–	–	–
18	F	MR negative	Not definitively discernible	Negative	Occipital	Unclear		–	–	–
44	M	DNET left mesiotemporal	Left temporal	lEft mesiotemporal	Left hemisphere	Left mesiotemporal	Anterior-temporal resection	DNET	Class 1	13
16	M	MR negative	Right temporal	Negative	Right temporal	Right temporal	AMTLR right	N/A	Class 1	14
16	F	FCD right frontal	Not definitively discernible	Bilateral temporal ventral	Temporal, not lateralisable	Right frontal		–	–	–
34	M	MR negative	Not discernible	Left insula dorsal	Left cingulum and insula	Left insula		–	–	–
36	M	MR negative	Not discernible	Right temporal	Right hemisphere	Right temporal		–	–	–
31	M	Cavernoma left frontal	No seizures recorded	Left frontal	Bilateral	Left frontal		–	–	–
15	M	MR negative	Left hemisphere	Negative	Left frontal	Left hemisphere		–	–	–
14	F	Bilateral perisylvic polymicrogyria	Bilateral temporal and temporo-parietal	Left mesial to temporal pole	Bilateral	Bilateral perisylvic polymicrogyria		–	–	–
22	F	MR negative	Not lateralisable	Right frontal precentral gyrus	Frontal, not lateralisable	Right frontal	Frontal lesionectomy right	FCD 2a	Class 1	20
14	F	Left gyrus frontalis sup. dysplasia	Left frontal/hemispheric	Negative	Left frontal	Left frontal	Lesionectomy dorsal gyrus frontalis superior left	FCD 2b	Class 1	15
28	F	MR negative	Right temporal lateral	Right temporomesial anterior	Right temporal	Right temporal	AMTLR right	FCD 2a	Class 2	17
27	F	MR negative	Multiregional	Negative	Multiregional	Unclear		–	–	–
32	M	MR negative	No seizures recorded	Negative	Frontal, not lateralisable	Unclear		–	–	–
15	M	MR negative	Right temporal	negative	Right temporal	Unclear	AMTLR right	Chronic epileptic changes of the HC	Class 2	9
28	M	MR negative	Right temporal	Negative	Right temporal	Right temporal	AMTLR right with amygdala hippocampectomy	Chronic epileptic changes with Chaslin-gliosis	–	–
18	F	MR negative	Right temporal	Right temporal, basal/lateral, central	right temporal	right temporal		–	–	–
45	M	Bilateral HC malrotation	Right temporal	Negative	Right temporal/insular	Bilateral HC	Temporal lobe resection right with amygdala hippocampectomy	Chronic epileptic changes with Chaslin-gliosis	–	–

where available, resected areas and histology results are presented as well. *AMTLR*  anteromedial temporal lobe resection, *DNET*  dysembryoplastic neuroepithelial tumor, *FCD*  focal cortical dysplasia, *MOGHE*  mild malformation of cortical development with oligodendroglial hyperplasia in epilepsy

### Comparison of 7 T MRI to clinical routine data

All MRI data were evaluated independently by two board-certified neuroradiologists specialized in epilepsy neuroimaging (rater 1: G.K., 16 years of neuroradiological experience in epilepsy neuroimaging; rater 2: L.H., two years of experience in epilepsy neuroimaging). First, they were blinded to clinical data and separately evaluated the 7 T data patient-wise, with a focal location hypothesis based on semiology made available to the raters only afterward. After a cool-down period of at least two weeks, the same process was repeated with clinical 3 T MRI. In a final round, 3 T and 7 T MRI were compared directly.

The evaluated parameters for 3 T and 7 T MRI were (1.) the presence of lesions (yes/no), (2.) the radiologist’s confidence in the identification of a present (scaled 1–3, with 3 being the highest), and (3.) its delineation (descriptive, detailed in Supp. Table 2). Imaging features denoted were blurred gray matter/white matter interface, transmantle signs, local signs of brain atrophy, abnormalities of the hippocampus and temporal lobe in general, as well as neoplasms and vascular abnormalities.

All completed ratings were aggregated by G.H., averaging confidence scores (with a 0 used in case a rater did not identify any lesion in a subject; rounded up) and summarizing 7 T delineation benefits compared to 3 T and to the clinical gold standard. For the presence of lesions, the detection rates for 3 T and 7 T, as well as inter-rater kappa (κ) for 3 T and 7 T, were calculated. Identification confidence between 3 and 7 T was tested using a paired Wilcoxon Signed-Rank tests.

We defined the final study outcome for 7 T MRI as percentages for:

1. Lesion identification as either “3 T MR-negative with 7 T lesion finding “ (also known in the literature as diagnostic gain), “7 T findings without plausible epileptogenicity”, or “both 3 T and 7 T MR-negative”.
2. Improvements in delineation in 3 T-positive cases as “7 T improved lesion delineation over 3 T”, “MR-positive at 3 T, but MR-negative at 7 T”, or “equivalent delineation at 3 T and 7 T”.
3. “Overall cases with benefits of 7 T MRI over 3 T MRI”, i.e., the sum of improved lesion identification and delineation over the whole cohort.
4. “Potentially positive treatment impact by 7 T”, i.e., the sum of improved lesion identification and delineation over the whole cohort, or “no new diagnostic information by 7 T” compared to the above-defined clinical gold standard. In patients that received surgery and had a follow up evaluation after three months, we identified patients that did not achieve seizure freedom as “surgical outcomes worse than ILAE class 1 with additional 7 T findings”.

## Results

### Cohort measurement

Forty-one patients were recruited, of whom 38 completed the 7 T protocol. One subject was excluded due to a previously undisclosed 7 T-unsafe cosmetic implant (after consent, but prior to scanning) and two subjects (one adolescent) aborted the 7 T scan due to stress regarding the enclosed space inside the scanner. In addition, only the expected transient, sensory side-effects of 7 T scans [22] (vertigo, discomfort with scan noises and enclosure, electro-gustatory perceptions) were reported. Figure 1 visualizes the participation process. The 38 remaining patients (age range 14–49 years, 20 females) are characterized in detail in Table 1. No data from the three dropout cases were processed further. The remaining subjects included mainly clinical 3 T MRN cases (n = 21), as well as those with insufficient clinical delineation (n = 17) necessary for surgical planning.

At the end of evaluation, 16 of the 38 patients had received surgery and pathohistological lesion evaluation (Table 1) while surgical outcome data (ILAE Outcome Classification) was available for 14 of these 16 patients (median of 22 months after surgery). In 12 of 16 histological classified patients, there was a previous 7 T finding. In 6/10 cases at 3 T and 7/11 cases at 7 T histological diagnosis was correctly predicted based on MRI alone.

### 7 T versus 3 T evaluation

When considering only 3 T MR imaging, rater #1 reported a positive detection rate of 45%, with an inter-rater κ of 0.66. In contrast, at 7 T, rater #1 had a detection rate of 55%, with a κ of 0.79. Considering all 22 patients with either 3 T or 7 T findings, mean confidence scores for 3 T (1.64 ± 0.84) were lower than for 7 T MRI (1.95 ± 1.19) (p = 0.050). These findings are displayed in detail in Table 2. Mean scores were higher for 7 T in nine patients, equal in ten, and higher for 3 T in three. At 7 T, relevant motion artifacts were found in two cases and B_1_^+^-inhomogeneities, especially of the right-side temporal lobe, were visible in all patients. As patient #6, who had only 3 T findings, did not receive surgery during data analysis, we could not clarify whether this signified worse performance at 7 T or a 3 T false-positive result.

**Table 2:** Results of 7 T MRI compared to 3 T MRI, including aggregated rater findings of lesion presence, confidence, and added value of 7 T scans versus 3 T MRI alone, as well as the full clinical gold standard

Patient	Agreggated ratings				7T added value	
	7T presence	3T Presence	7T Confidence	3T Confidence	Overall 7T compared to 3T	Overall 7T compared to gold standard
1	Yes/Yes	Yes/Yes	2.5	2.5	Delineation and microstructural changes better visible at 7T	Improved delineation and microstructure
2	Yes/Yes	Yes/Yes	2	**2.5**	Better delineation at 7T, especially SWI, but dysplasia better visible at 3T	Improved delineation
3	No/No	No/No	–	–	3T and 7T MRN	7T MRN
4	No/No	No/No	–	–	3T and 7T MRN	7T MRN
5	Yes/Yes	Yes/Yes	3	3	3T and 7T comparable, but TMS better visible at 7T	Slightly improved visibility
6	No/No	Yes/No	0	**0.5**	Only visible at 3T	Only visible at 3T
7	No/No	No/No	–	-	3T and 7T MRN	7T MRN
8	Yes/Yes	Yes/Yes	2	**3**	Better delineation and impression of gliosis over TMS compared to 3T	Better delineation and other characterization
9	No/No	No/No	–	–	3T and 7T MRN	7T MRN
10	No/No	No/No	–	–	3T and 7T MRN	7T MRN
11	No/No	No/No	–	–	3T and 7T MRN	7T MRN
12	No/No	No/No	–	–	3T and 7T MRN	7T MRN
13	No/No	No/No	–	–	3T and 7T MRN	7T MRN
14	Yes/Yes	Yes/Yes	3	3	Better delination, but different signal alteration at 7T	Improved delineation
15	Yes/Yes	Yes/Yes	3	3	Better resolution of internal HC structure	Better resolution of internal HC structure
16	Yes/Yes	Yes/Yes	2	2	Equivalent confidence, basal B1+ inhomogeneity as issue	No diagnostic improvement
17	Yes/Yes	No/No	**1**	0	Lesion visible, but only identified with gold standard information	Only identified with gold standard information
18	Yes/Yes	Yes/Yes	3	3	Improved delineation of cortical malformation, especially on SWI	Improved delineation of cortical malformation, especially on SWI
19	No/No	No/No	-	–	3T and 7T MRN	7T MRN
20	Yes/Yes	No/No	**2**	0	More microbleeds visible at 7T, global assymmetry visible at 7T	No diagnostic improvement
21	Yes/Yes	No/Yes	**2**	1.5	TMS better visible, HC alterations only visible at 7T	Improved lesion detection
22	Yes/Yes	Yes/Yes	3	3	Better delineation at 7T, but no comparison data for DNETs at 7T	Improved delineation
23	No/No	No/No	–	–	3T and 7T MRN	7T MRN
24	Yes/Yes	No/Yes	**2**	1.5	Better delineation at 7T	Improved delineation
25	No/No	No/No	–	–	3T and 7T MRN	7T MRN
26	Yes/No	No/No	**1**	0	HC malformation visible	HC malformation identified
27	Yes/Yes	Yes/Yes	3	3	Better Delineation of vessels in SWI	Improved delineation
28	No/No	No/No	–	–	3T and 7T MRN	7T MRN
29	Yes/Yes	Yes/Yes	**2**	1.5	Higher confidence and greater visible lesion extend	Higher confidence and greater visible lesion extend
30	Yes/No	Yes/Yes	**2**	0.5	Better delineation	Better delineation
31	Yes/No	Yes/No	1	1	More detailed visualization	More detailed visualization
32	Yes/Yes	No/No	**1**	0	HC malformation visible, but impacted by B1+ inhomogeneity	HC malformation identified
33	Yes/No	No/No	**1**	0	HC malformation visible	HC malformation identified
34	No/No	No/No	–	–	3T and 7T MRN	7T MRN
35	No/No	No/No	–	–	3T and 7T MRN	7T MRN
36	No/No	No/No	–	–	3T and 7T MRN	7T MRN
37	No/No	No/No	–	–	3T and 7T MRN	7T MRN
38	Yes/No	Yes/No	1.5	1.5	Better resolution of internal HC structure	Better resolution of internal HC structure
**Overall**	** κ = 0.79**	**κ = 0.66**	**1.95 ± 1.19 **	**1.64 ± 0.84 **		

*HC*  hippocampus, *SWI*  susceptibility-weighted imaging, *TMS*  transmantle sign

### 7 T delineation benefits

**Fig. 3:**
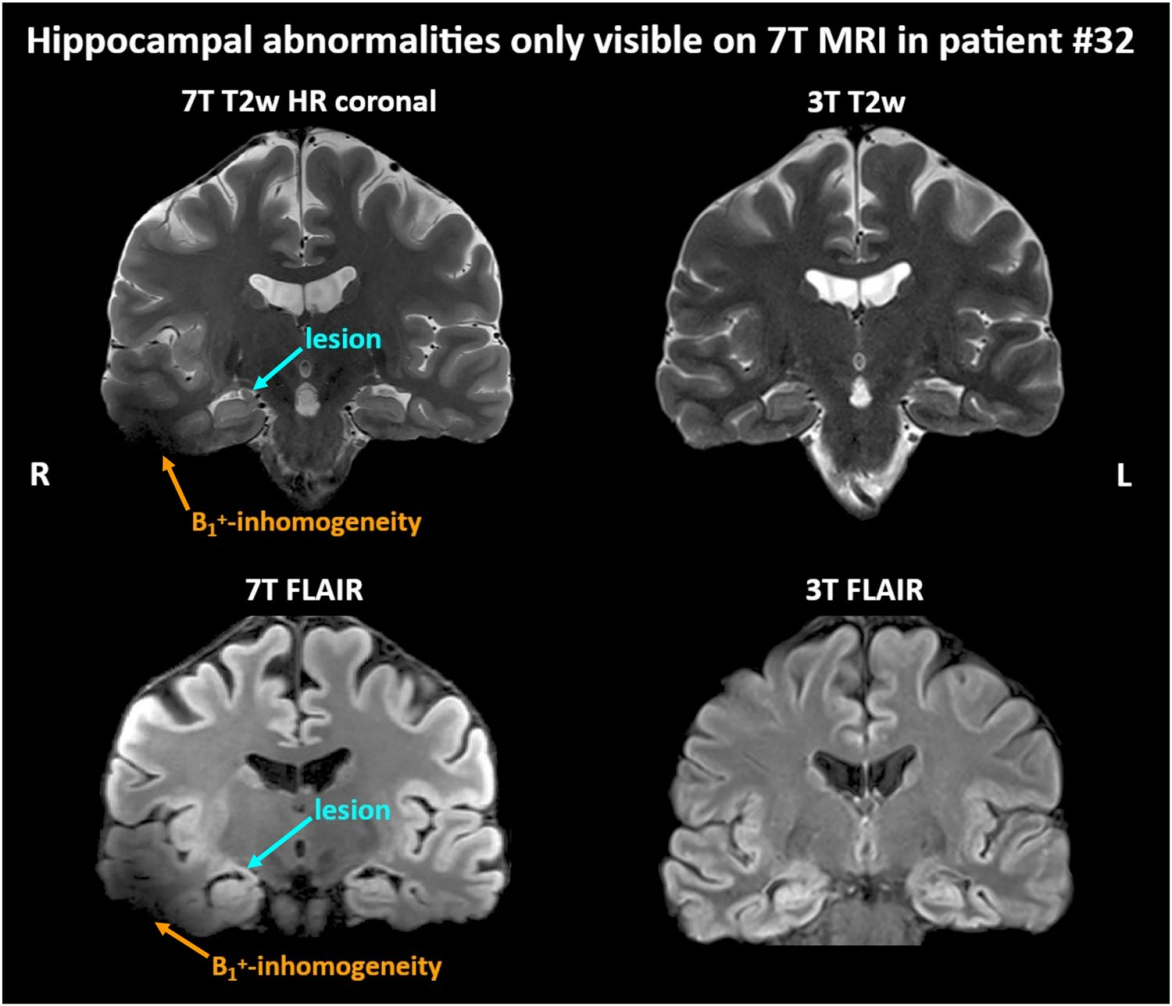
In this case, patient #32, 7 T MRI could identify hippocampal asymmetry regarding size and structure of the right hippocampus whereas 3 T MRI remained negative. On T2w 7 T MRI, the right hippocampus appeared larger during neuroradiological evaluation than the left side. Arrows highlight regions of interest for pathology or artifacts. *T2w*  T2-weighted, *FLAIR*  Fluid-attenuated inversion recovery, *7 T*  7 Tesla, *3 T*  3 Tesla, *HR*  high resolution, *B1*^+^  radio frequency excitation, *R* right side, *L* left side

**Fig. 4:**
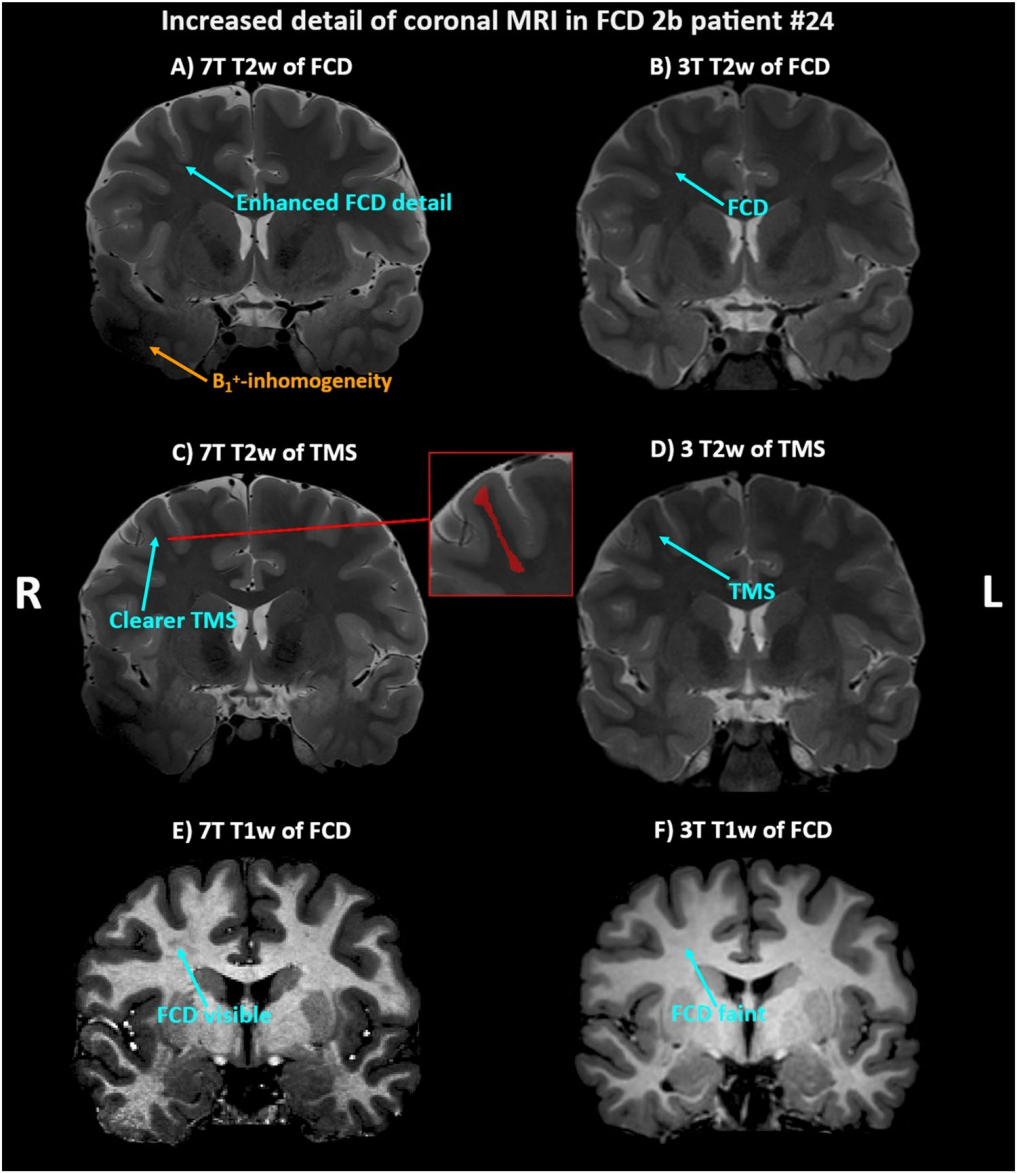
In patients such as #24, the 7 T epilepsy consensus protocol adds visible details that could be useful for neurosurgical planning, such as the enhanced delineation of an focal cortical dysplasia (FCD) (**A**) vs. **B**)) or the accompanying trans-mantle sign (TMS) in image (**C**) vs. **D**)) of this example. On T1w imaging (**E** vs **F**), the FCD is clearly visible at 7 T, while inconspicuous at 3 T. Arrows highlight regions of interest for pathology or artifacts. *T1w*  T1-weighted, *T2w*  T2-weighted, *7 T* 7 Tesla, *3 T* 3 Tesla, *B1*^+^  radio frequency excitation, *R* right side, *L* left side

**Fig. 5:**
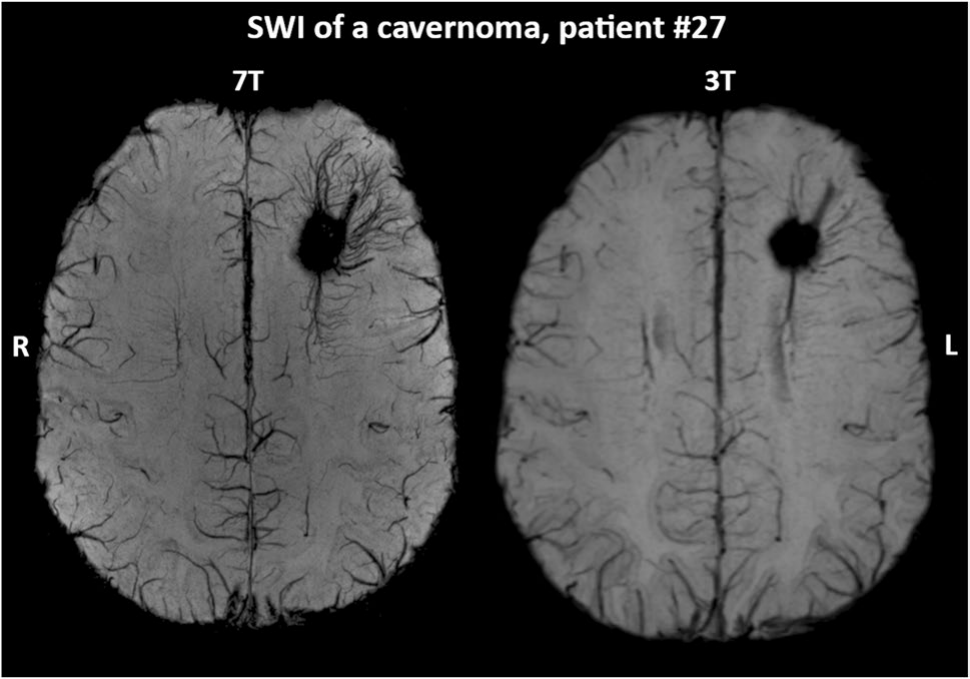
Example of the ability of 7 T susceptibility-weighted imaging (SWI) to enhance the imaging of vessels compared to 3 T in skull-stripped transversal images of patient #27. There is a clear increase of detail in the delineation of the developmental venous anomaly surrounding the cavernoma. *7 T* 7 Tesla, *3 T* 3 Tesla, *R* right side, *L* left side

We observed specific improvements offered by the 7 T Epilepsy Task Force consensus protocol over clinical 3 T MRI. The new findings in 3 T MRN patients included three cases of structural hippocampal abnormalities and one of FCD located in the temporal lobe. Figure 3 shows one of these cases, where 7 T MRI identified an abnormal right hippocampus. In 15 of 17 cases (88%) with 3 T MRI findings, the 7 T protocol added new information. This included better delineation of lesion extent based on better visibility of abnormalities, i.e., the transmantle sign (TMS), cortical dysplasia, or microgyria (Fig. 4). In particular, 7 T SWI performed better in identifying blood vessel abnormalities that are sometimes associated with cortical malformations, as seen in Fig. 5. Overall, the raters considered T2w coronal MRI most important due to its high resolution. These findings are all summarized in Table 2, with the full rater-wise evaluation available in Supp. Table 2.

### Overall 7 T MRI benefits within the cohort

Of the twenty-one 3 T MRN patients, four (19%) patients were “3 T MR-negative with 7 T lesion finding “, one (5%) patient had a “7 T finding without plausible epileptogenicity”, and the remaining 16 (76%) patients remained “both 3 T and 7 T MR-negative”.

Of the 17 patients with 3 T findings, 15 (88%) patients had “7 T improved lesion delineation”, “equivalent confidence at 3 T and 7 T” in one case, and “MR-positive at 3 T, but MR-negative at 7 T” in one case (6%).

In summary, 19 of 38 cases (50%), were rated as “overall cases with benefits of 7 T MRI over 3 T MRI” compared to 3 T MRI and with a “potentially positive treatment impact by 7 T” compared to the clinical gold standard. In the remaining 50%, we found “no new diagnostic information by 7 T”. We found “surgical outcomes worse than ILAE Class 1 with additional 7 T findings” in two out of four cases. These results are summarized in Table 3.

**Table 3:** Summary of results and added benefits of the 7 T consensus protocol to clinical data as defined in the Methods section. In summary, in our cohort although only 19% of 3 T MRN cases had a relevant 7 T finding, 88% of 3 T identified lesions could be better delineated using 7 T images. Compared to the clinical standard of clinical onset monitoring, 3 T MRI, PET, and EEG, in half of the patients, 7 T MRI was judged to potentially lead to a positive impact on treatment

7 T comparison to 3 T	Number	Percentage
*Presence*		
3 T MR-negative with 7 T lesion finding	4/21	19%
7 T finding without plausible epileptogenicity	1/21	5%
Both 3 T and 7 T MR-negative	16/21	76%
*Delineation*		
7 T improved lesion delineation over 3 T	15/17	88%
MR-positive at 3 T but MR-negative at 7 T	1/17	6%
Equivalent delineation at 3 T and 7 T	1/17	6%
*Overall*		
Cases with benefits of 7 T MRI	19/38	50%
Comparison to clinical gold standard		
Potentially positive treatment impact by 7 T	19/38	50%
No new diagnostic information by 7 T	19/38	50%
Surgical outcomes worse than ILAE Class 1 with additional 7 T findings	2/4	50%

## Discussion

Our study clearly demonstrates evidence that the newly recommended 7 T Epilepsy Task Force consensus protocol provided potential radiological benefits in 50% of our patient cohort. We were able to identify previously undetected lesions in 19% of 3 T MRN patients and found relevant additional lesion information in 88% of 3 T-visible abnormalities at 7 Tesla imaging with a higher but non-significant radiological confidence than at 3 T. This indicates that the addition of the 7 T MRI consensus protocol to routine clinical practice in presurgical epilepsy investigation using powerful 3 T MRI protocols could be primarily beneficial to the characterization and delineation of relevant pathological entities. Moreover, the 50-min protocol implemented during our study was feasible with only two aborted scan sessions.

### Comparison to the state of research

Compared to the expectations set by the consensus protocol publication [10], i.e., better delineation but unlikely new identification of FCDs, more apparent hippocampal abnormalities, better LEAT characterization, and the importance of SWI for vascular malformations, we found high agreement within our cohort. This also includes the identification of B1 + inhomogeneity and subject motion as negative impacts on the utility of 7 T images. Van Lanen et al. [16], who pooled 16 studies that included 275 patients of sufficient design quality, found diagnostic gains defined as 8% to 67% percent of subjects in individual studies who were 7 T-positive, but lower-field-negative. Their reported diagnostic gain of 31% over all studies was higher than the 19% in our single-center study, but that gain reflects larger patient numbers and included clinical 1.5 T MRI as well as differing 7 T protocols.

A second recently published review [17] has described a lesion detection rate of 22% for 3 T MRI and 65% for 7 T MRI in a subset of analyzed studies. Comparing these observations to our different overall rates of findings of 45% for 3 T and 55% for 7 T also demonstrates the importance of a well-defined patient population. Feldman et al., for example, found a higher rate of 7 T lesion detection in MRN cases (25 of 37)[23], but 24 of these patients had clinical 1.5 T scans as a reference in a study cohort size similar to our own, producing higher 7 T benefits than a comparison to state-of-the-art clinical 3 T MRI. De Ciantis et al. [24] investigated a mixed cohort of 1.5 T/3 T MRN cases and found lesions on 7 T MRI in 29% of patients. Verseema et al. [25] observed epileptogenic lesions on 7 T MRI in 23% of 40 mostly 3 T MRN patients. Similar results were seen by Wang et al. [26] with a 22% lesion detection rate in sixty-seven 3 T MRN patients, which is very close to our findings. Interestingly, they improved this result to 43% by the application of morphometric analysis software. Colon et al. [27] investigated 19 3 T MRN cases with 7 T lesion detection in three (16%). Using aditional guidance by magnetoencephalography, they could also identify lesions in another three cases (16%).

Van Lanen et al. published their intent to conduct a more standardized 7 T MRI study [28], and even plan to go beyond the acquisition of T1/T2/T2*-contrast images by including diffusion tensor imaging (DTI) and arterial spin labeling (ASL) in their 7 T protocols.

Future research could include advanced 7 T metabolic imaging modalities, such as CEST [29] or MRSI [30] to yield new insights into MRN epilepsy. But while metabolic changes in other brain pathologies, such as gliomas, can easily be correlated to clinical imaging [31], verification of findings in MRN epilepsy requires significant analytic work and clinical confirmation.

We conclude that the availablility of clinical 3 T MRI versus 1.5 T MRI is the primary driving factor in determining the percieved benefits of 7 T MRI and should, therefore, be discussed when comparing 7 T epilepsy imaging studies. Our study was performed in the setting of a busy tertiary care epilepsy surgery program with experienced epilepsy imaging specialists as readers. This is important, as many existing studies in the field have used general radiological or clinical neurological readers as a reference and their results may have been changed after thorough analysis by an epilepsy imaging expert [32]. As the variety of findings from hippocampal abnormalities to FCDs in our results show, the strength of the consensus protocol lies in the coverage of multiple possible etiologies when other tools of preclinical evaluation remain inconclusive.

### Limitations

As our current 7 T scanner is without clinical certification, we could not use our findings in order to modify neurosurgical planning. Thus, no comment on the benefit of 7 T MRI regarding surgical outcome can be made. Whether the additional 7 T findings in two of four surgical failures would have influenced results remains speculative. Our cohort size was small and heterogeneous, making conclusions challenging, especially considering that histological confirmation as the ground-truth was available only for 16 patients and surgical outcome regarding seizure status only in 14. We maintain that our cohort reflects the clinical reality faced in many tertiary epilepsy centers. Exploring 7 T MRI in epilepsy further will require multi-center studies that combine large cohorts with homogeneous inclusion criteria, standardized 7 T approaches such as the one tested in our study, and most importantly, a clear definition of clinical diagnostic gold standards, including histology and surgical outcome.

Our 7 T images show the need for parallel transmit technologies [12, 33] to address field inhomogeneities and reliably measure the temporal lobe, which is highly relevant for epilepsy diagnostics. Furthermore, motion artifacts in multiple scans did negatively affect image quality and should be avoided with motion detection and correction methods in the future [34].

## Conclusion

We demonstrated a successful implementation of the 7 T Epilepsy Task Force consensus recommendation for pre-surgical evaluation in a cohort of 38 pharmaco-resistant patients with refractory focal epilepsies. Using the new consensus protocol, our results should be readily pool-able with future studies that follow the same standards. We, therefore, see our presented work as pilot data for future multi-center studies with standardized evaluation.

Ultimately, we demonstrated the utility of the 7 T consensus protocol in a clinically relevant setting that reflects realizable clinical benefits and found potentially epileptogenic lesions in 19% of 3 T MRN cases, and offered more detailed information than 3 T MRI in 88% of the other cases. Therefore, morphological 7 T imaging for epilepsy should not be overemphasized. Nonetheless, in 50% of cases where surgery achieved a worse ILAE classification than class 1, only 7 T MRI detected any abnormalities. In total, 7 T MRI had a potentially beneficial effect over 3 T MRI and other clinical diagnostics in half the study cohort.

### Supplementary Information

Below is the link to the electronic supplementary material.

Supplementary file1 (XLSX 39 KB)

## Data Availability

Pseudonymized 7 T image data from this study cohort will be made available by request from any qualified investigator after approval of the Medical University of Vienna data clearing commission.

## Abbreviations

3 T: 3 Tesla
7 T: 7 Tesla
B1^+^: Radio frequency excitation
DNET: Dysembryoplastic neuroepithelial tumor
FCD: Focal cortical dysplasia
FDG: Fluorodeoxyglucose
FLAIR: Fluid-attenuated inversion recovery
GG: Ganglioglioma
HCS: Hippocampal sclerosis
HR: High resolution
ILAE: International league against epilepsy
L: Left side
LEAT: Epilepsy-associated tumor
MET: Methionine
MRI: Magnetic resonance imaging
MRN: MR-negative
MUV: Medical University of Vienna
R: Right side
PET: Positron emission tomography
SWI: Susceptibility-weighted imaging
T1w: T1-weighted
T2w: T2-weighted
T2*w: T2*-weighted
TMS: Transmantle sign
WM: White matter
WMS: White matter suppressed
