# Correction to: Implementation of a 7T Epilepsy Task Force consensus imaging protocol for routine presurgical epilepsy work-up: effect on diagnostic yield and lesion delineation

**DOI:** 10.1007/s00415-024-12257-9

**Published:** 2024-04-05

**Authors:** Gilbert Hangel, Gregor Kasprian, Stefanie Chambers, Lukas Haider, Philipp Lazen, Johannes Koren, Robert Diehm, Katharina Moser, Matthias Tomschik, Jonathan Wais, Fabian Winter, Vitalij Zeiser, Stephan Gruber, Susanne Aull-Watschinger, Tatjana Traub-Weidinger, Christoph Baumgartner, Martha Feucht, Christian Dorfer, Wolfgang Bogner, Siegfried Trattnig, Ekaterina Pataraia, Karl Roessler

**Affiliations:** 1 Department of Neurosurgery, Medical University of Vienna, Währinger Gürtel 18-20, 1090 Vienna, Austria; 2 Department of Biomedical Imaging and Image-Guided Therapy, High Field MR Centre, Medical University of Vienna, Vienna, Austria; 3 Christian Doppler Laboratory for MR Imaging Biomarkers, Vienna, Austria; 4 Medical Imaging Cluster, Medical University of Vienna, Vienna, Austria; 5 Division of Neuroradiology and Musculoskeletal Radiology, Department of Biomedical Imaging and Image-Guided Therapy, Medical University of Vienna, Vienna, Austria; 6 NMR Research Unit, Faculty of Brain Science, Queens Square MS Centre, Department of Neuroinflammation, UCL Queen Square Institute of Neurology, University College London, London, United Kingdom; 7 Department of Radiology and Nuclear Medicine, VU University Medical Centre, Amsterdam, The Netherlands; 8 Department of Neurology, Klinik Hietzing, Vienna, Austria; 9 Center for Rare and Complex Childhood Onset Epilepsies, Member of ERN EpiCARE, Department of Pediatrics and Adolescent Medicine, Medical University of Vienna, Vienna, Austria; 10 Department of Neurology, Medical University of Vienna, Vienna, Austria; 11 Division of Nuclear Medicine, Department of Biomedical Imaging and Image-Guided Therapy, Medical University of Vienna, Vienna, Austria

**Correction to: Journal of Neurology (2024) 271:804–818** 10.1007/s00415-023-11988-5

In the original version of this article, Table 1 has been duplicated as Table 2. The correct Table 2 is given in the following page.

**Table 2:** Results of 7 T MRI compared to 3 T MRI, including aggregated rater findings of lesion presence, confidence, and added value of 7 T scans versus 3 T MRI alone, as well as the full clinical gold standard

Patient	Agreggated ratings				7 T added value	
	7 T presence	3 T presence	7 T confidence	3 T confidence	Overall 7 T compared to 3 T	Overall 7 T compared to gold standard
1	Yes/yes	Yes/yes	2.5	2.5	Delineation and microstructural changes better visible at 7 T	Improved delineation and microstructure
2	Yes/yes	Yes/yes	2	**2.5**	Better delineation at 7 T, especially SWI, but dysplasia better visible at 3 T	Improved delineation
3	No/no	No/no	–	–	3 T and 7 T MRN	7 T MRN
4	No/no	No/no	–	–	3 T and 7 T MRN	7 T MRN
5	Yes/yes	Yes/yes	3	3	3 T and 7 T comparable, but TMS better visible at 7 T	Slightly improved visibility
6	No/no	Yes/no	0	**0.5**	Only visible at 3 T	Only visible at 3 T
7	No/no	No/no	–	–	3 T and 7 T MRN	7 T MRN
8	Yes/yes	Yes/yes	2	**3**	Better delineation and impression of gliosis over TMS compared to 3 T	Better delineation and other characterization
9	No/no	No/no	–	–	3 T and 7 T MRN	7 T MRN
10	No/no	No/no	–	–	3 T and 7 T MRN	7 T MRN
11	No/no	No/no	–	–	3 T and 7 T MRN	7 T MRN
12	No/no	No/no	–	–	3 T and 7 T MRN	7 T MRN
13	No/no	No/no	–	–	3 T and 7 T MRN	7 T MRN
14	Yes/yes	Yes/yes	3	3	Better delination, but different signal alteration at 7 T	Improved delineation
15	Yes/yes	Yes/yes	3	3	Better resolution of internal HC structure	Better resolution of internal HC structure
16	Yes/yes	Yes/yes	2	2	Equivalent confidence, basal B1 + inhomogeneity as issue	No diagnostic improvement
17	Yes/yes	No/no	**1**	0	Lesion visible, but only identified with gold standard information	Only identified with gold standard information
18	Yes/yes	Yes/yes	3	3	Improved delineation of cortical malformation, especially on SWI	Improved delineation of cortical malformation, especially on SWI
19	No/no	No/no	–	–	3 T and 7 T MRN	7 T MRN
20	Yes/yes	No/no	**2**	0	More microbleeds visible at 7 T, global assymmetry visible at 7 T	No diagnostic improvement
21	Yes/yes	No/yes	**2**	1.5	TMS better visible, HC alterations only visible at 7 T	Improved lesion detection
22	Yes/yes	Yes/yes	3	3	Better delineation at 7 T, but no comparison data for DNETs at 7 T	Improved delineation
23	No/no	No/no	–	–	3 T and 7 T MRN	7 T MRN
24	Yes/yes	No/yes	**2**	1.5	Better delineation at 7 T	Improved delineation
25	No/no	No/no	–	–	3 T and 7 T MRN	7 T MRN
26	Yes/no	No/no	**1**	0	HC malformation visible	HC malformation identified
27	Yes/yes	Yes/yes	3	3	better delineation of vessels in SWI	Improved delineation
28	No/no	No/no	–	–	3 T and 7 T MRN	7 T MRN
29	Yes/yes	Yes/yes	**2**	1.5	Higher confidence and greater visible lesion extend	Higher confidence and greater visible lesion extend
30	Yes/no	Yes/yes	**2**	0.5	Better delineation	Better delineation
31	Yes/no	Yes/no	1	1	More detailed visualization	More detailed visualization
32	Yes/yes	No/no	**1**	0	HC malformation visible, but impacted by B1 + inhomogeneity	HC malformation identified
33	Yes/no	No/no	**1**	0	HC malformation visible	HC malformation identified
34	No/no	No/no	–	–	3 T and 7 T MRN	7 T MRN
35	No/no	No/no	–	–	3 T and 7 T MRN	7 T MRN
36	No/no	No/no	–	–	3 T and 7 T MRN	7 T MRN
37	No/no	No/no	–	–	3 T and 7 T MRN	7 T MRN
38	Yes/no	Yes/no	1.5	1.5	Better resolution of internal HC structure	Better resolution of internal HC structure
**Overall**	**κ = 0.79**	**κ = 0.66**	**1.95 ± 1.19**	**1.64 ± 0.84**		

*HC* hippocampus, *SWI* susceptibility-weighted imaging, *TMS* transmantle sign

The original article has been corrected.

